# Evaluating Aspects of Online Medication Safety in Long-Term Follow-Up of 136 Internet Pharmacies: Illegal Rogue Online Pharmacies Flourish and Are Long-Lived

**DOI:** 10.2196/jmir.2606

**Published:** 2013-09-10

**Authors:** Andras Fittler, Gergely Bősze, Lajos Botz

**Affiliations:** ^1^Department of PharmaceuticsMedical SchoolUniversity of PecsPecsHungary

**Keywords:** online pharmacies, Internet pharmacy, online pharmaceutical services, online medicines, counterfeit medicines, patient safety

## Abstract

**Background:**

A growing number of online pharmacies have been established worldwide. Among them are numerous illegal websites selling medicine without valid medical prescriptions or distributing substandard or counterfeit drugs. Only a limited number of studies have been published on Internet pharmacies with regard to patient safety, professionalism, long-term follow-up, and pharmaceutical legitimacy verification.

**Objective:**

In this study, we selected, evaluated, and followed 136 Internet pharmacy websites aiming to identify indicators of professional online pharmacy service and online medication safety.

**Methods:**

An Internet search was performed by simulating the needs of potential customers of online pharmacies. A total of 136 Internet pharmacy websites were assessed and followed for four years. According to the LegitScript database, relevant characteristics such as longevity, time of continuous operation, geographical location, displayed contact information, prescription requirement, medical information exchange, and pharmaceutical legitimacy verification were recorded and evaluated.

**Results:**

The number of active Internet pharmacy websites decreased; 23 of 136 (16.9%) online pharmacies ceased operating within 12 months and only 67 monitored websites (49.3%) were accessible at the end of the four-year observation period. However, not all operated continuously, as about one-fifth (31/136) of all observed online pharmacy websites were inaccessible provisionally. Thus, only 56 (41.2%) Internet-based pharmacies were continuously operational. Thirty-one of the 136 online pharmacies (22.8%) had not provided any contact details, while only 59 (43.4%) displayed all necessary contact information on the website. We found that the declared physical location claims did not correspond to the area of domain registration (according to IP address) for most websites. Although the majority (120/136, 88.2%) of the examined Internet pharmacies distributed various prescription-only medicines, only 9 (6.6%) requested prior medical prescriptions before purchase. Medical information exchange was generally ineffective as 52 sites (38.2%) did not require any medical information from patients. The product information about the medicines was generally (126/136, 92.6%) not displayed adequately, and the contents of the patient information leaflet were incomplete in most cases (104/136, 76.5%). Numerous online operators (60/136, 44.1%) were defined as rogue Internet pharmacies, but no legitimate Internet-based pharmacies were among them. One site (0.7%) was yet unverified, 23 (16.9%) were unapproved, while the remaining (52/136, 38.2%) websites were not available in the LegitScript database. Contrary to our prior assumptions, prescription or medical information requirement, or the indication of contact information on the website, does not seem to correlate with “rogue pharmacy” status using the LegitScript online pharmacy verification standards. Instead, long-term continuous operation strongly correlated (*P*<.001) with explicit illegal activity.

**Conclusions:**

Most Internet pharmacies in our study sample were illegal sites within the definition of “rogue” Internet pharmacy. These websites violate professional, legal, and ethical standards and endanger patient safety. This work shows evidence that online pharmacies that act illegally appear to have greater longevity than others, presumably because there is no compelling reason for frequent change in order to survive. We also found that one in five websites revived (closed down and reopened again within four years) and no-prescription sites with limited medicine and patient information are flourishing.

## Introduction

The Internet has revolutionized communication, trade, and health services. Three-quarters of EU households have access to the Internet and one-third of Europeans used the Internet on mobile devices away from home or work [1]. One of the most popular uses of the Internet is to find medical information [2] and thus many patients rely on it as their main resource [3]. Although the Web offers numerous opportunities to improve health, it also presents an enormous health hazard since it represents an unregulated market with almost no consumer protection [4]. Internet-based commerce provides access to a large variety of health-related products, such as complementary medicines, over-the-counter medications (OTC), and prescription-only medicines via Internet-based pharmacies (also called online pharmacies, cyber pharmacies, or e-pharmacies) [5].

Online pharmacies can be beneficial to consumers (eg, convenience, privacy, free access to information, comparison shopping, etc) but can also carry with them numerous disadvantages (eg, lack of meaningful interaction with physician and pharmacists, misdiagnosis, inappropriate use of medicines, personal data protection, etc) [5-7]. These disadvantages and dangers are further exacerbated in the case of unlicensed and illegally operated online pharmacies.

Regrettably, the majority of online drug sellers violate safe pharmacy practices and laws, as numerous websites sell medicines without valid medical prescriptions and have been shown to distribute substandard, illegal, unapproved, or counterfeit drugs [4,6,8]. The likelihood of encountering such potentially dangerous medicines on the Internet is extremely high as the number of illegally operating online pharmacy sites is enormous [6] and legitimate sellers are crowded out by illicit websites [9]. In fact, more than one hundred million Web pages are indexed in Google when searching “no prescription required” [10] and out of more than 35,000 evaluated active pharmacy websites 95.0% were classified as not legitimate according to the LegitScript Internet pharmacy certification standards [11]. The latest report by the National Association of Boards of Pharmacy (NABP) also found that the vast majority of Internet pharmacy sites are operating in contravention of US federal and state pharmacy laws, as 96.7% of more than 10,000 reviewed Internet drug outlets selling prescription medications were identified as “not recommended” [12].

In many cases, customers are not aware that products offered by online pharmacies may not have the same quality that a retail pharmacy may offer [13,14] and often it is difficult to determine whether a website is legitimate or not [15], further making consumer differentiation between an original drug and a counterfeit version a difficult task [16]. Consequently, the growth of the unregulated drug market and the spread of counterfeit medications are becoming a serious public health risk [17,18], while also creating a financial burden on the health care system due to ineffective drug treatments, adverse events, and needed remedial care [9,17,19].

Although different classifications for online pharmacies have been published [6, 20-23], from the patient safety perspective, Internet-based pharmacies can be classified basically as (1) legitimate Internet pharmacy websites providing high-quality pharmacy services according to verification standards, and (2) illegitimate online pharmacies that are not verified and may not comply with national or international professional standards and regulations. Legitimate Internet pharmacies must adhere to both the laws and regulations of the country where the website operates from and the destination country [24]. These websites are verified, monitored, and require valid prescriptions for prescription-only medicines; thus, they are safe to use and can be recommended for patients. Illegitimate online pharmacies either have not been subject to a certification process or fail to comply with national or international standards and regulations. Such illegal online vendors may sell medicines without prescriptions or market counterfeit and substandard products [6,17]. Accordingly, the safety of illegitimate websites is not assured and these Internet pharmacies should not be recommended for patients. Illicit Internet pharmacies can further be divided into different subcategories based on how drugs are prescribed and dispensed. The term “rogue” Internet pharmacy (although often used interchangeably with “illegal”) refers to websites that facilitate the sale of drugs in a way that is not subject to sufficient regulatory oversight [24]. These rogue online vendors enable the sale of prescription drugs or other medicinal products (either directly on the website or indirectly by directing patients to another website) and violate applicable laws or regulations [24]. Those websites that provide drugs without a legitimate prescription from a physician are often titled “no-prescription online pharmacies” [25]. It is most likely that individuals who may be behind rogue websites are licensed neither as pharmacies nor as pharmacists [26]. Illegitimate websites often operate from developing countries with lack of regulatory oversight and enforcement regarding Internet-based commercial operations [27].

Owing to the principles of free movement of goods, capital, and services, especially in the European Union [28], but also in other parts of the world due to free-trade agreements and customs unions and the anonymous always-changing nature of the Internet, the illegal online sale of medicines is difficult to regulate. In fact, it is probably impossible to keep the Internet free from illegal sites [13,29]. A global approach and international legal framework with adequate safety standards is needed to regulate online medical services [29-31]. Until a globally accepted and effective method is developed, it is important to evaluate and study online pharmacies as useful “snapshots” of this problematic issue.

Several papers have been published on the evaluation of online pharmacies and products obtained from such sources. Numerous important findings are summarized in a comprehensive review by Orizio et al on products offered, prescription requirement, online questionnaires, prices, drug information, etc [5]. Most studies confirm that the majority of online pharmacies do not require customers to possess a valid prescription [32,33] nor any medical information from patients [34]. Furthermore, patients have insufficient access to relevant medication information when ordering drugs online [35]. Despite the complexity of this issue and the different methods used for the evaluation of online pharmacies, consumer/patient safety is a commonly evaluated parameter of most studies [5]. Although numerous characteristics of online pharmacies can be evaluated, it is of great importance to determine specific parameters that may be considered as reliable indicators of trustworthy online pharmacies.

We have hypothesized that the quality of service is strongly related to the increasing age of the pharmacy, as illegal operators are either eliminated due to market forces driven by the public intervention of unsatisfied customers or as a consequence of law enforcement and legal regulations [22]. The requirement for a valid medical prescription is a widely studied parameter of online pharmacies [5] and its absence was considered to be a potential indicator of illegal activity. In addition, we believed that professional certifications are the most specific indicators of high-quality health content and medication safety, thus pharmacy accreditation logos and legitimacy verifications—such as Internet pharmacy classifications according to the LegitScript database—are important indicia of valid online pharmacy operations. Consequently, in this study we selected, evaluated, and followed 136 Internet pharmacy websites for four years, aiming to identify indicators of professional online pharmacy service and online medication safety.

## Methods

### Overview

A specific evaluation tool was developed, including questions regarding the longevity, contact information, and the geographical location of online pharmacies. Also, we documented the distributed product categories, requirement of medical prescriptions and health questionnaires, channels of information exchange, and patient safety (see Multimedia Appendix 1). Correlations between operational longevity (age effect), distributing medicines without valid prescriptions (prescription requirement), availability of drug information and online consultation (information exchange), street address and telephone number (contact information), and legitimacy certifications (approval by LegitScript) were assessed to evaluate potential indicators of medication safety.

Websites were identified using the Google search engine; the Internet search was performed with the aim of finding websites selling one or more of 15 previously selected active ingredients. The active ingredients were chosen arbitrarily by the inclusion of regularly used and popular pharmacological categories, based on the authors’ professional experience, literature results, and pharmaceutical consumption databases. We aimed to simulate needs of different potential customers of online pharmacies; thus, a wide variety of drug classes were selected. We have included in our study anti-obesity drugs (sibutramine, orlistat) and medications used in erectile dysfunction (sildenafil), as the earlier Internet pharmacies tended to sell principally lifestyle medications [5]. An opioid analgesic (tramadol), two commonly used anxiolytics (diazepam, alprazolam), two antidepressants (venlafaxine, fluoxetine), and two nonsteroid anti-inflammatory drugs (diclofenac, acetaminophen/paracetamol) were also included. One commonly used antibacterial for systemic use (amoxicillin) and a systemic antiviral (oseltamivir) were also among our searched medicines. Although most medications used without medical control can have unfavorable consequences, corticosteroids for systemic use (methylprednisolone, prednisolone) and an antineoplastic agent (methotrexate) were also analyzed, as their inappropriate use is fraught with danger.

Online pharmacies were searched for using the search engine Google with the keywords “buy”, “online”, and “pharmacy”, with the combination of the name of the active ingredient listed above (eg, “buy sibutramine”, “sibutramine online”, or “sibutramine pharmacy” as specific search terms). By documenting the first 20 references appearing in Google, we could simulate what patients can easily find and what websites they most probably visit when searching for online pharmacies, since 95% of search engine users click on the hits/records displayed on the first two search engine results pages [36]. Websites with multiple hits were included once in our database. Only websites operating in English were included in our study, as it was supposed that they are available to a broad clientele worldwide.

A total number of 300 records were gathered and screened between February and March 2008, which led to a database of 136 websites, excluding duplicates and websites that did not sell products. Numerous relevant professional characteristics of online pharmacies such as the longevity (time of existence) of the online pharmacy, location of operation, product categories sold, requirement of medical prescription, medical information exchange, payment and delivery conditions, user-friendliness, and applicability were recorded and evaluated by the authors (see Multimedia Appendix 1 for the complete list of questions). The data was summarized by descriptive analysis and a chi-square test was performed to evaluate correlations between legitimacy according to LegitScript verification database and potential indicators of medication safety (longevity, prescription requirement, information exchange, and displayed contact information).

### Long-Term Accessibility of Online Pharmacies

The longevity of Internet pharmacies was assessed by documenting the online availability of the website’s domain name between February 2008 and February 2012 at 6-10 month interim periods. An Internet pharmacy was considered to be active if the pharmacy website was accessible and inactive if it was not available. Continuity of operation was calculated through the first disappearance (unavailability). Those websites that were available throughout the whole study interval of 48 months were considered as continuously operational, while those that were not available at one or more occasions but reopened again at some time were defined as reviving Internet pharmacies.

### Location of Operation and Contact Information

Identification and accessibility of the medicine supplier is of significant importance as the anonymity provided by the Internet allows online pharmacies to conceal the street address and the telephone number of their companies. We aimed to gather data on the availability of contact information on the website and the actual geographical location of the server according to its IP address.

Each website was evaluated by the assessment of the main page, the copyright information, the contact information, the frequently asked questions, and the “about us” pages. We documented the year the site was established and any contact information (street address, telephone number) stated by the operators. The IP address was acquired by the “tracert” command in Microsoft Windows and the geographical location was determined by a free Web-based program available at www.IP2location.com.

### Product Categories Offered

We assessed the variety (brand or generic) and types (OTC, prescription-only) of drugs sold and also how many active ingredients were offered by the online pharmacy website. During the evaluation of the ordering process, the billing, shipping, and payment options were documented.

### Requirement of Medical Prescription

During the ordering and the payment process, the authors documented if the website requested medical prescriptions for prescription-only medicines. Countries may define pharmaceuticals differently according to prescription requirement but, since legitimate Internet pharmacies must also adhere to the regulations of the country the website offers to ship drugs to, we have taken Hungarian regulations into account and considered only two nonsteroid anti-inflammatory drugs (diclofenac and acetaminophen) as OTC.

### Exchange of Medical Information

In our survey, we evaluated how online pharmacies gather data from their customers (information requirement) and also analyzed the quantity and quality of medical information patients can find (information availability). The authors evaluated whether the websites used online questionnaires or whether there was an opportunity to interact with the provider and/or health care professionals on the website via online chat, telephone, or email. The available medical information regarding the pharmaceuticals marketed was determined by assessing the general product information (introducing the medication and its effects) during the ordering process. We have also evaluated the online version of the patient information leaflet, which contains specific information about medical conditions, doses, side effects, storage, pregnancy, breast-feeding, etc. Both sources of medication information were categorized as (1) “detailed” if the authors judged the provided data to be enough to support safe use of the product, (2) “incomplete” when only a short description of the affect was highlighted or important sections of the patient information leaflet were missing, and (3) “not available” if no data was accessible regarding the medication.

### Accreditation and Verification of Internet Pharmacy Websites

Several codes and seals of verification can be displayed on online pharmacy websites. In addition to different nonpharmaceutical verification methods (eg, TRUSTe logo confirming the identity of the company and VeriSign logo guaranteeing privacy principles and data protection), specific professional accreditation programs exist for Internet pharmacies in some countries like the United States (Verified Internet Pharmacy Practice Site or VIPPS [37], which is recommended by the US Food and Drug Administration, and LegitScript [11] whose standards are accepted by the US National Association of Board of Pharmacy), United Kingdom (Registered Internet Pharmacy [38] under the UK General Pharmaceutical Council), or Germany (German Institute of Medical Information and Documentation or DIMDI [39], which registers official pharmacies approved for a mail order permit of sales). In addition, private certification services without government sanction or recommendation also exist such as the Canadian International Pharmacy Association (CIPA) or PharmacyChecker. We searched for pharmacy-related private and professional organizational logos and seals on the main page of active websites at the end of the observation period.

### Legitimacy According to LegitScript Verification Database

LegitScript is a private company monitoring online pharmacies; its pharmacy certification program adheres to NABP recognized standards [23]. Currently, the company maintains the world’s largest database of Internet pharmacies and health-related websites, more than 35,000 and 290,000, respectively [11]. To evaluate the legitimacy of online pharmacy websites included in our research, we checked the URL addresses within the LegitScript database at the end of the four-year observation period in 2012. Internet pharmacy websites are classified by LegitScript into four categories (legitimate, unverified, unapproved, and rogue). “Legitimate” sites are those that have been through a verification process and are confirmed to meet LegitScript standards. “Unverified” Internet pharmacies are likely to comply or easily able to comply, with minimum adjustments to LegitScript requirements, but have not been subject to the certification process. “Unapproved” Internet pharmacies do not comply with LegitScript’s verification standards or applicable laws or regulations, but do not meet the definition of being rogue. “Rogue” Internet pharmacies are those illegitimate websites that directly or indirectly facilitate the sale of prescription drugs or other medicinal products and violate, appear to violate, or encourage violation of US federal or state law or regulation. These online vendors do not adhere to accepted standards of medicine and/or pharmacy practice (including standards of safety) and/or are engaged in fraudulent or deceptive business practices. Although LegitScript maintains a large database of websites, the ones that were not evaluated by the organization were labeled as “Not available in the database” in our study. Offline websites remain in the LegitScript database until the domain name registration expires.

In the United States, the NABP operates VIPPS, a voluntary accreditation program that lists Internet pharmacies either as accredited or “not recommended”. The latter are Internet drug sellers that do not comply with state and federal laws and regulations and NABP’s criteria assuring patients’ health and safety [8]. The VIPPS accreditation was not chosen as a primary indicator, since to date NABP has reviewed a smaller sample of 10,000 sites and VIPPS is subject to application and annual participation fees.

## Results

### Long-Term Accessibility of Online Pharmacies

**Figure 1:**
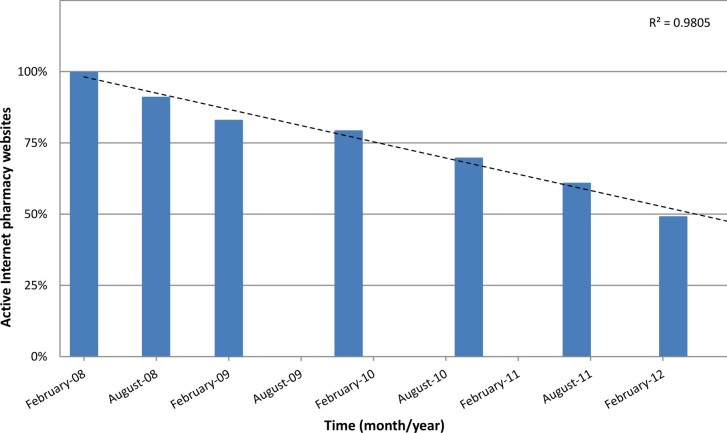
Percentage of active websites (including revival sites) from the original study sample of 136 Internet pharmacies.

The number of active Internet-based pharmacy websites evaluated in our study was constantly decreasing (Figure 1). Within one year, 23 of 136 (16.9%) websites disappeared or ceased operation, while just 67 (49.3%) monitored Internet pharmacy websites were accessible at the end of the four-year observation period from the original sample of 136. The number of operating Internet pharmacies decreases with time; this trend shows a negative correlation (linear regression *R* ^2^= 0.9805). It is important to note that not all of the 67 websites operated continuously during the four years. We observed that about one-fifth (31/136) of all observed online pharmacy websites “closed down” (were inaccessible) at least once and became active again during the four-year time span (ie, revived). Only 56 of the 136 (41.2%) Internet-based pharmacies were in continuous operation for the four-year study period.

### Location of Operation and Contact Information

Thirty-one (22.8%) of the 136 online pharmacies did not display any contact information (postal address or telephone number), while only 59 (43.4%) displayed both pieces of information on the website. Although most Internet pharmacies (102/136, 75.0%) showed their telephone number for customers, only 63 (46.3%) gave details of the physical location on their website. Seventy-three (53.7%) of the 136 Internet pharmacies did not give information on postal address; 25 sites stated they were located in Europe (18.4%), 19 in North America (14.0%), 5 in the Pacific (3.7%), 4 in the Caribbean (2.9%), and 10 elsewhere (7.3%).

To test online pharmacy location claims, we compared the website’s declared physical location with the area of its domain registration according to the IP address. We found that the declared physical location did not correspond to the area of domain registration for most websites, as only 32 (23.5%) of 136 Internet pharmacy domains were registered in the same continent as the declared physical location of operation and only 13 (9.6%) sites operated their server within the borders of the same country. According to the identification of the IP address, website domains were most frequently located in the following countries: United States (69/136, 50.7%), United Kingdom (14/136, 10.3%), Russia (9/136, 6.6%), Panama (7/136, 5.1%), Israel (5/136, 3.7%), Canada (4/136, 2.9%), Netherlands (4/136, 2.9%), New Zealand (4/136, 2.9%), and 20 (20/136, 14.7%) elsewhere.

### Product Categories Offered

In general, online pharmacies in our study sample specialized in selling prescription only medicines (120/136, 88.2%) and few offered over-the-counter medications exclusively (16/136, 11.8%). Most Internet pharmacies (72/136, 52.9%) sell only generic drugs; roughly one-fifth (26/136, 19.1%) offered solely brand name products. Herbal, hygiene products, and cosmetics were infrequently offered in our sample.

With respect to the types of different active ingredients sold, online pharmacies divided themselves into two main categories. The great majority (111/136, 81.6%) were vendors of more than 10 different active substances. Lifestyle drugs (eg, sildenafil and orlistat) and opioid painkillers (eg, codeine and tramadol) were generally available on almost every website. Others (13/136, 9.6%) focused on marketing only one therapeutic, for example, tramadol or sibutramine. Further, it took only a couple of minutes to find doxorubicin, erythropoietin, or even stem cells marketed over the Internet.

### Requirement for Medical Prescriptions

Similar to previous earlier reports of online medicine sales without valid medical prescriptions [32,33,40], here only 9 (6.6%) of the 136 online pharmacies in this study requested them. The remaining 127 (93.4%) allowed checkout (the last step of online shopping process) and credit card payment without any medical documentation.

### Exchange of Medical Information

Online medical questionnaires were the major source of information on the health status of patients (84/136, 61.8%) but 52 sites (38.2%) did not require any medical information from customers before buying medicines. Sixty-six (48.5%) of the 136 studied online pharmacies made online consultation available on their website via online chat, telephone, or email (Table 1).

The availability of medical information was also limited on most websites (Table 2). General product information (medical use, description of pharmacological effect, and indications) was generally (126/136, 92.6%) not adequately displayed. Most sites may assume that customers know what they need and that people are making decisions exclusively based on product price. The patient information leaflet was found to be detailed on just one website (0.7%); it was incomplete in most cases (104/136, 76.5%), and the authors were not able to identify all its essential chapters on nearly every fourth website (31/136, 22.8%).

### Accreditation and Verification of Internet Pharmacy Websites

At the end of the four-year observation period, we examined the 67 active Internet pharmacies and we could identify eight types of pharmacy-related seals posted on 11 (16.4%) websites. These included CIPA and PharmaCheck seals displayed on 3 websites each, TrustedRx and PharmacyChecker logo found on 2 sites each, and a Registered Internet Pharmacy (UK) seal on one website. A vast majority (56/67, 83.6%) of active study online pharmacies did not present any pharmaceutical or professional logo. The VIPPS or LegitScript legitimacy seals were not observed in our study sample.

### Legitimacy According to LegitScript Verification Database

We checked the URL addresses of the online pharmacies in this study using the LegitScript database. Out of the 136 Internet pharmacies evaluated in our sample, 52 (38.2%) websites were not available in the database, 1 site (0.7%) was yet unverified, while 23 online pharmacies (16.9%) were unapproved. Numerous online operators (60/136, 44.1%) were defined as rogue Internet pharmacies, but no legitimate Internet-based pharmacies were among them. Table 3 shows the distribution of Internet pharmacies of different legitimacy codes within all evaluated sites and websites that were continuously operational for four years. As can be seen, the vast majority of long-lived websites are unsafe for patients, as they are either unapproved (17/56, 30.4%) or rogue (36/56, 64.3%).

The correlation between rogue online pharmacy legitimacy category and certain important characteristics of Internet pharmacies (longevity, prescription requirement, medical questionnaire, and contact information) was analyzed to evaluate which properties may indicate illegitimate operation or refer to patient health risks (Table 4). Our results did not support our initial hypothesis that rogue online pharmacies operate for a shorter time period. On the contrary, more rogue (36/56, 64.3%) online pharmacies operated continuously for 48 months. Somewhat less than half (57/127, 44.9%) of the websites not requiring medical prescriptions from the total study sample were rogue pharmacies. Similar results were obtained when the requirement of medical information in a medical questionnaire was evaluated, as out of 52 Internet pharmacy sites dispensing medicines without requesting customers to provider their medical status on an online form, 23 (44.4%) were rogue. Highlighting the physical location or the telephone number of the online pharmacy on the website did not seem to affect verification category (Table 4). Thus, only the time of continuous operation correlated (negatively) with legitimacy using LegitScript verification database.

**Table 1:** Sources of medical information exchange on Internet pharmacy websites (N=136).

		n (%)
**Online consultation**		
	Available	66 (48.5)
	Not available	70 (51.5)
**Self-completed online medical questionnaire**		
	Required	84 (61.8)
	Not required	52 (38.2)

**Table 2:** Information availability on marketed medicines (N=136).

		n (%)
**General product information**		
	Detailed	2 (1.5)
	Incomplete	8 (5.9)
	Not displayed	126 (92.6)
**Patient information leaflet**		
	Detailed	1 (0.7)
	Incomplete	104 (76.5)
	Not displayed	31 (22.8)

**Table 3:** LegitScript legitimacy code of total evaluated Internet pharmacies and continuously operational websites.

LegitScript legitimacy code categories	Evaluated Internet pharmacy websites, n (%)	Websites within the “48 months continuously operational” category, n (%)
Legitimate	0 (0)	0 (0)
Unverified	1 (0.7)	1 (1.8)
Unapproved	23 (16.9)	17 (30.4)
Rogue	60 (44.1)	36 (64.3)
Not available in the database	52 (38.2)	2 (3.6)
Total	136 (100)	56 (100)

**Table 4:** Correlation between various evaluated factors of 136 online pharmacies with rogue Internet pharmacy verification status according to LegitScript database.

Selected outcome parameters of our online evaluation process (n)		Rogue^a^	Not defined as rogue^b^	Chi-square test
**Continuous long-term operation**				
	Yes (56)	36 (64.3)	20 (35.7)	*P*<.001
	No (80)	24 (30.0)	56 (70.0)	
**Prescription requirement**				
	Yes (9)	3 (33.3)	6 (66.7)	*P*=.50
	No (127)	57 (44.9)	70 (55.1)	
**Requirement of medical information in an online questionnaire**				
	Yes (84)	37 (44.0)	47 (56.0)	*P*=.98
	No (52)	23 (44.2)	29 (55.8)	
**Geographical location is stated on website**				
	Yes (62)	28 (45.2)	34 (54.8)	*P*=.82
	No (74)	32 (43.2)	42 (56.8)	
**Telephone number indicated on website**				
	Yes (102)	47 (46.1)	55 (53.9)	*P*=.43
	No (34)	13 (38.2)	21 (61.8)	

^a^Rogue Internet pharmacies are websites that directly or indirectly facilitate the sale of prescription drugs or other medicinal products, and violate, appear to violate, or encourage violation of Federal or state law or regulation.

^b^Not defined as rogue online pharmacy category includes unapproved (that do not meet verification standards), unverified (not evaluated) online pharmacies, and websites not found in the LegitScript database.

## Discussion

### Principal Findings

Our longitudinal study of Internet pharmacies shows that the longevity of operations seems to be associated with illegal status and one-fifth of the websites revived during the four years. Further, the majority of websites are no-prescription Internet pharmacies and the limited availability of patient information and opportunity for patient discussions are negative determinants of patient and medication safety on many of these websites.

A great number of online pharmacies operate illegally and offer medicines to buyers without a valid medical prescription. However, of importance, they offer these illicit sales long term. As far as we know, this study provides the first evidence that, in fact, illegal activities are correlated with longevity success in the illicit online pharmacy market. However, with virtually all of these online sellers highly suspect because they provide little substantive information and incomplete patient information, they create patient safety risks. Perhaps even more importantly, they may subject their buyers to the risks of counterfeit, poor quality, and dangerous forms of medications without medical supervision.

In the longitudinal study of the survival of 136 Internet pharmacy websites, we searched the Internet with the key words “buy”, “online”, “pharmacy”, together with the combination of the names of 15 active ingredients. Numerous relevant professional characteristics of online pharmacies were recorded and evaluated (see Multimedia Appendix 1 for complete list of questions). It was observed that several (23/136, 16.9%) online pharmacies closed down within the first year while only half of them (67/136, 49.3%) were accessible at the end of the four-year observation period. We noticed that not all of these remained active continuously; more than one-fifth (31/136, 22.8%) of online pharmacies included in our study were classified as a “revival site”, where it ceased its continuous activity transitionally but resumed operation sometime later. Only 56 (41.2%) Internet-based pharmacies were in continuous operation while the remaining ones (80/136, 58.8%) were inaccessible at least one time during the four-year study period. It is also interesting to note that more than one-third (31/80, 38.8%) of these closed down websites revive. Thus, it is likely that more reviving websites could have been identified with more regular (possibly weekly or monthly) visits during follow-up.

These reviving sites may aim to temporarily disappear to avoid actions of legal authorities or unsatisfied customers. We have also observed that domain names of inactive websites are advertised. These domains are presumably sold to other Internet pharmacy operators who may take advantage of having well-known brand names or ones that rank high with search engines.

It is clear that the Internet and its support organizations allow entities to conceal their street address and the telephone number of their actual operators. Consequently, it is not surprising that less than half of the online pharmacies displayed their contact street address on their website. In fact, websites may fraudulently present false information to attract their customers. For example, it has been reported that patients and potential customers who find Canadian online pharmacies more trustworthy buy products from websites displaying Canadian symbols, but in reality the website may be registered elsewhere and the products originate from other countries [41]. In our study, we found that the declared physical location (displayed on the website) did not correspond to the registration domain (according to IP address) for the majority of the studied Internet pharmacies, as only one of 10 online pharmacies’ domain name registration was within the country of operation as declared on the website.

Although it would be difficult to draw unambiguous conclusions from these findings, it is a warning sign that numerous Internet pharmacies veil their contact information, while ones providing such details generally register their domains in countries other than displayed. Such great discordance between the location of domain registration compared with physical location may indicate that online pharmacies mainly operate from remote countries.

This may be an important warning sign; for example, the World Health Organization (WHO) has noted medicines purchased over the Internet from illegal websites that conceal their physical address have been found to be counterfeit in over 50% of cases [18].

Similar to other work, we found that few online pharmacies requested prescriptions and sub-optimum online consultations or questionnaires are the primary way to conduct information transfer. As befits the requirement for medical prescriptions, patients should have access to quality information regarding the risks, benefits, and optimal use of their medications. Similarly, consumers must partner with health care providers by sharing information about their health status, other medications, allergies, and other potential interactions to assist providers to help patients reach their health care goals.

The approaches used by existing and reviving websites would likely not fulfill these basic patient needs. Indeed, online questionnaires have been reported to be inadequate tools to assess the health status of consumers, aiming more at giving consumers a false sense of health assurance rather than performing an effective assessment of health status [40]. Further, these questionnaires de-emphasize the real nature and risks of drugs [34]. Ultimately, there is no guarantee that the online consultation (either chat, email, or telephone) is actually provided by a licensed physician or pharmacist, as the identity and licensure status of the professional who makes or reviews the prescription is usually not revealed [5].

These risks to patients from illicit online vendors are exacerbated by the limited general product information and incomplete patient information leaflets. One of the greatest dangers of obtaining medicines online is the potential health hazard from lack of professional information exchange and face-to-face communications between patients and health professionals. Fundamentally, patients cannot make informed decisions about the safe and appropriate use of their medications [35]. The risk of holding back relevant medical information or providing false data in online interactions is significantly higher than during traditional patient-physician consultations.

Today, it is almost impossible to keep the Internet free of illegal sites as the traditional laws that regulated the prescribing process previously are ineffective in regulating international Internet drug sales [13]. Urgent steps are required to combat the unregulated online sale of medicines and to protect people purchasing drugs from the Internet. Governmental and professional bodies are currently developing national and internationally harmonized regulations (eg, Ryan Haight Online Pharmacy Consumer Protection Act in the United States [42] or Directive 2011/62/EU of the European Parliament and of the Council in Europe [43]) and focusing on public campaigns regarding the dangers of online medicines and counterfeit drugs (eg, US Food and Drug Administration Consumer update on The Possible Dangers of Buying Medicines over the Internet [44] and the BeSafeRx national campaign [45]). We believe that adequate regulatory environment, effective law enforcement, and raising public awareness are key elements of safeguarding online medication safety.

Several professional organizations have developed accreditation/verification systems for Internet pharmacy websites to improve patient safety by distinguishing reliable websites from illegal operations. For example, the Health On the Net Foundation certification (HONcode) is an ethical standard aiming to offer quality health information [46], as well as specific pharmacy-based systems in the United Kingdom [38] and Germany [39], and cooperative public agency-accepted systems such as VIPPS [37]. Such verification systems seem to be promising solutions to help customers find legitimate websites and safe medicines because websites go through strict accreditation processes assessing licensure, facilities, personnel, privacy rules, etc. However, these are also limited as they are currently voluntary and, due to the scarcity or virtual absence of accredited vendors [47], patients are most likely to find rogue sites when browsing the Internet. Further, accreditation logos can also be misleading due to the unauthorized use of legitimacy seals [47] or because illegal operators can display fake seals or verification logos.

Likely none of these measures alone will be effective enough to combat illicit marketing and sales, as the vast majority of Internet pharmacy sites are currently rogue. The NABP’s “.pharmacy” generic top-level domain is a promising initiative as the proposed new domain suffix would be restricted to legitimate pharmacies and other prescription drug-related organizations worldwide [12].

We believe that it would also be beneficial if search engines could more effectively set back illicit websites in their search result pages and simultaneously favor recommended Internet pharmacy websites in their search algorithms. Indeed, customers of online pharmacies are vulnerable to fraud and it is most likely that the majority of patients cannot differentiate illegal sites from legal online operations; for example, even university students with education in health sciences do not make appropriate judgments about health information provided on the Internet [19]. Accordingly, another possibility is to focus on the potential customers themselves and describe safe ways to purchase medicines over the Internet develop a methodology for patients to evaluate the safety and quality of online pharmacy sites. The distribution of medicines without valid prescriptions, limited contact information on the website, and poor channels of information exchange (medical questionnaires, drug information) could potentially be negative indicators of online medication safety. It should be noted that these parameters did not reach significance in this study. However, it is likely that combined evaluation of various parameters together with longevity may provide an effective tool (see factors listed in Multimedia Appendix 1).

### Limitations

Our study does have some limitations. We did not actually purchase any drugs; however, we believe that the evaluation of Web pages alone can indicate numerous signs of danger. Further, compared to the tremendous number of existing Internet pharmacy websites, we searched, evaluated, and followed a relatively small sample at various intervals. Furthermore, we accessed the data at 6-10 month intervals; by visiting websites more frequently, more accurate data could presumably have been gathered on longevity and revival activity. Finally, the abundance of illegitimate sellers and the regrettable fact that legitimate Internet pharmacies do not rank high on the search engine result pages (or inversely, illicit websites are not set back effectively by the search engine) resulted in not having any LegitScript “legitimate” approved sites in our study sample; although we performed what we believe are typical searches, our results may reflect an over-inclusion of illicit rather than LegitScript legitimate websites.

### Conclusions

Overall, this work shows evidence that online pharmacies that act illegally appear to have greater longevity than others. We also found that one in five websites revive and no-prescription sites with limited medicine and patient information are flourishing. The findings suggest that illegitimate operators can provide fraudulent online services and disregard safe pharmacy standards without legal or commercial consequences worldwide. Consequently, a more effective international legislation and enforcement is needed to battle the complex globalized market of illegal vendors.

## Multimedia Appendix 1

Website evaluation tool for the assessment and follow-up of Internet pharmacies.

## Abbreviations

CIPA: Canadian International Pharmacy Association
HON: Health On the Net Foundation
HONcode: HON Foundation code of conduct
NABP: National Association of Boards of Pharmacy
OTC: over-the-counter medications
VIPPS: Verified Internet Pharmacy Practice Site
WHO: World Health Organization

## References

[1] Seybert H (2012). Eurostat Statistics in focus 50/2012.

[2] Seybert H (2011). Eurostat Statistics in focus 66/2011.

[3] Andreassen HK, Bujnowska-Fedak MM, Chronaki CE, Dumitru RC, Pudule I, Santana S, Voss H, Wynn R (2007). European citizens' use of E-health services: a study of seven countries. BMC Public Health.

[4] Levaggi R, Orizio G, Domenighini S, Bressanelli M, Schulz PJ, Zani C, Caimi L, Gelatti U (2009). Marketing and pricing strategies of online pharmacies. Health Policy.

[5] Orizio G, Merla A, Schulz PJ, Gelatti U (2011). Quality of online pharmacies and websites selling prescription drugs: a systematic review. J Med Internet Res.

[6] Liang BA, Mackey T (2009). Searching for safety: addressing search engine, website, and provider accountability for illicit online drug sales. Am J Law Med.

[7] Fittler A, Lanko E, Brachmann B, Botz L (2012). Behaviour analysis of patients who purchase medicines on the internet: can hospital pharmacists facilitate online medication safety?. European Journal of Hospital Pharmacy: Science and Practice.

[8] National Association of Boards of Pharmacy www.nabp.net.

[9] Liang BA, Mackey TK (2012). Sexual medicine: Online risks to health--the problem of counterfeit drugs. Nat Rev Urol.

[10] Google Google Hungary.

[11] LegitScript (2013). www.legitscript.com.

[12] National Association of Boards of Pharmacy (2013). www.nabp.net.

[13] Montoya ID, Jano E (2007). Online pharmacies: safety and regulatory considerations. Int J Health Serv.

[14] Fittler A, Bosze G, Botz L (2010). [Attitude of patients and customers toward on-line purchase of drugs--a Hungarian survey by community pharmacies]. Orv Hetil.

[15] Jackson G, Patel S, Khan S (2012). Assessing the problem of counterfeit medications in the United Kingdom. Int J Clin Pract.

[16] Siva N (2010). Tackling the booming trade in counterfeit drugs. Lancet.

[17] Mackey TK, Liang BA (2011). The global counterfeit drug trade: patient safety and public health risks. J Pharm Sci.

[18] World Health Organization (2012). www.who.int.

[19] Ivanitskaya L, Brookins-Fisher J, O Boyle I, Vibbert D, Erofeev D, Fulton L (2010). Dirt cheap and without prescription: how susceptible are young US consumers to purchasing drugs from rogue internet pharmacies?. J Med Internet Res.

[20] Jena AB, Goldman DP, Foster SE, Califano JA (2011). Prescription medication abuse and illegitimate internet-based pharmacies. Ann Intern Med.

[21] Mäkinen MM, Rautava PT, Forsström JJ (2005). Do online pharmacies fit European internal markets?. Health Policy.

[22] Arruñada B (2004). Quality safeguards and regulation of online pharmacies. Health Econ.

[23] National Association of Boards of Pharmacy (2013). www.nabp.net.

[24] LegitScript (2013). www.legitscript.com.

[25] Cicero TJ, Ellis MS (2012). Health outcomes in patients using no-prescription online pharmacies to purchase prescription drugs. J Med Internet Res.

[26] National Association of Boards of Pharmacy (2013). www.nabp.net.

[27] Baert B, De Spiegeleer B (2010). Quality analytics of Internet pharmaceuticals. Anal Bioanal Chem.

[28] European Union The (2012). The official Journal of the European Union C 326.

[29] Liang BA, Mackey TK (2012). Vaccine shortages and suspect online pharmacy sellers. Vaccine.

[30] Seeberg-Elverfeldt NJ (2009). Mail-order trade in medicines in Europe--a guide for legislators to protect consumers. Eur J Health Law.

[31] Liang BA, Mackey TK, Lovett KM (2012). Suspect online sellers and contraceptive access. Contraception.

[32] Raine C, Webb DJ, Maxwell SR (2009). The availability of prescription-only analgesics purchased from the internet in the UK. Br J Clin Pharmacol.

[33] Orizio G, Schulz P, Domenighini S, Caimi L, Rosati C, Rubinelli S, Gelatti U (2009). Cyberdrugs: a cross-sectional study of online pharmacies characteristics. Eur J Public Health.

[34] Orizio G, Rubinelli S, Schulz PJ, Domenighini S, Bressanelli M, Caimi L, Gelatti U (2010). "Save 30% if you buy today". Online pharmacies and the enhancement of peripheral thinking in consumers. Pharmacoepidemiol Drug Saf.

[35] Bessell TL, Anderson JN, Silagy CA, Sansom LN, Hiller JE (2003). Surfing, self-medicating and safety: buying non-prescription and complementary medicines via the internet. Qual Saf Health Care.

[36] Enge E, Spencer S, Fishkin R (2012). The Art of SEO.

[37] National Association of Boards of Pharmacy (2011). www.vipps.nabp.net.

[38] General Pharmaceutical Council www.pharmacyregulation.org.

[39] German Institute of Medical Documentation and Information DIMDI www.dimdi.de.

[40] Orizio G, Schulz P, Domenighini S, Bressanelli M, Rubinelli S, Caimi L, Gelatti U (2009). Online consultations in cyberpharmacies: completeness and patient safety. Telemed J E Health.

[41] Veronin MA (2007). Canadian Internet pharmacies: price, policy, and perspective. Res Social Adm Pharm.

[42] United States Congress H.R. 6353-110th Congress.

[43] European Parliament Council Of The European Union (2011). The official Journal of the European Union.

[44] U.S. Food and Drug Administration www.fda.gov.

[45] U.S. Food and Drug Administration www.fda.gov.

[46] Boyer C, Baujard V, Geissbuhler A (2011). Evolution of health web certification through the HONcode experience. Stud Health Technol Inform.

[47] Liang BA, Mackey TK (2012). Online availability and safety of drugs in shortage: a descriptive study of internet vendor characteristics. J Med Internet Res.

