# Supplementary material for: Evaluating Aspects of Online Medication Safety in Long-Term Follow-Up of 136 Internet Pharmacies: Illegal Rogue Online Pharmacies Flourish and Are Long-Lived
Source: J Med Internet Res. 2013 Sep 10;15(9):e199. doi: 10.2196/jmir.2606 (PMC3785996; doi:10.2196/jmir.2606)
Supplement: Supplementary file 1 [file jmir_v15i9e199_app1.pdf]

Supplementary File

Website evaluation tool for the assessment and follow-up of Internet pharmacies

Question category	Documented characteristics of online pharmacies	Variable
Identification of the operator	Domain name	URL
	Year website was established	date (year)
	Declared physical location of operation	world region & country & city
	Telephone contact	telephone number
	Location of the server according to IP address	world region & country
Longevity	Functionality at the actual time of observation	yes, if website is accessible
	Continuous operation for 4 years	yes, if accessible at all observations
	Reviving website	yes, if a functional website was inaccessible at any given time earlier
Products sold	Variety of drugs sold	brand / generic / both
	Type of drugs sold	prescription-only / over the counter medicines / both
	Number of active ingredients	number
Prescription requirement	Requirement of prior medical prescriptions for prescription-only drugs	yes / no
Information exchange	Availability of online consultation	yes / no
	Requirement of patient's medical information in a questionnaire	yes / no
	Availability of general product information	detailed / incomplete / not available
	Availability of the patient information leaflet	detailed / incomplete / not available
Payment and	Available payment methods (e.g. credit	number of methods

delivery	card, money transfer, PayPal)	
	Delivery time	days
	Cost of delivery	US dollars
	Refund policy	refund guarantee / no refund
User friendliness and appearance (subjective elements)	User friendly navigation	easy to use / medium / difficult to navigate
	General appearance and design	excellent / average / poor
	Customer feedback on website	displayed / not available
Legitimacy verification	Existence of legitimacy seal	type of logo
	Functionality of online logo	active link / inactive link
	Legitimacy according to LegitScript verification database	legitimate / unverified, unapproved / rogue / not available in the database
